# Voluntary workplace genomic testing: wellness benefit or Pandora’s box?

**DOI:** 10.1038/s41525-021-00276-8

**Published:** 2022-01-20

**Authors:** Kunal Sanghavi, Betty Cohn, Anya E. R. Prince, W. Gregory Feero, Kerry A. Ryan, Kayte Spector-Bagdady, Wendy R. Uhlmann, Charles Lee, J. Scott Roberts, Debra J. H. Mathews

**Affiliations:** 1 The Jackson Laboratory for Genomic Medicine, Farmington, CT USA; 2 Berman Institute of Bioethics, Johns Hopkins University, Baltimore, MD USA; 3 University of Iowa College of Law, Iowa City, IA USA; 4 Maine-Dartmouth Family Medicine Residency, Augusta, ME USA; 5 Center for Bioethics and Social Sciences in Medicine, University of Michigan, Ann Arbor, MI USA; 6 Department of Obstetrics & Gynecology, Center for Bioethics and Social Sciences in Medicine, University of Michigan, Ann Arbor, MI USA; 7 Department of Internal Medicine, Division of Genetic Medicine; Department of Human Genetics, University of Michigan School of Medicine, Ann Arbor, MI USA; 8 Precision Medicine Center, The First Affiliated Hospital of Xi’an Jiaotong University, 277 West Yanta Rd., Xi’an, 710061 Shaanxi China; 9 Department of Health Behavior and Health Education, School of Public Health, University of Michigan, Ann Arbor, MI USA; 10 Department of Genetic Medicine, Johns Hopkins University, Baltimore, MD USA

**Keywords:** Personalized medicine, Medical ethics

## Abstract

Consumer interest in genetic and genomic testing is growing rapidly, with more than 26 million Americans having purchased direct-to-consumer genetic testing services. Capitalizing on the increasing comfort of consumers with genetic testing outside the clinical environment, commercial vendors are expanding their customer base by marketing genetic and genomic testing services, including testing for pharmacogenomic and pathogenic variants, to employers for inclusion in workplace wellness programs. We describe the appeal of voluntary workplace genomic testing (wGT) to employers and employees, how the ethical, legal, and social implications literature has approached the issue of genetic testing in the workplace in the past, and outline the relevant legal landscape. Given that we are in the early stages of development of the wGT market, now is the time to identify the critical interests and concerns of employees and employers, so that governance can develop and evolve along with the wGT market, rather than behind it, and be based on data, rather than speculative hopes and fears.

Consumer interest in genetic and genomic testing is growing rapidly, with more than 26 million Americans having purchased direct-to-consumer (DTC) genetic testing services^1^. The global consumer genetic testing market is anticipated to exceed $2.5 billion by 2025^1–4^. Capitalizing on the increasing comfort of consumers with genetic testing outside the clinical environment, commercial vendors are expanding their customer bases by marketing genetic and genomic testing services, including testing for pharmacogenomic and pathogenic variants, to employers for inclusion in workplace wellness programs. For the purposes of this paper, voluntary workplace genomic testing (wGT) refers to *both* genetic and genomic testing offered by vendors through voluntary workplace wellness programs, as the full range of testing offered through such programs has not been robustly studied to date. Workplace wellness programs commonly include features like health risk assessments, biometric testing and screening, smoking cessation programs, and gym and health-club memberships^5^. We describe the potential appeal of wGT to employers and employees, how the ethical, legal, and social implications (ELSI) literature has approached the issue of workplace genetic testing in the past, and outline the current relevant legal landscape in the United States (US). Given that we are in the early stages of development of the wGT market, now is the time to identify the critical interests and concerns of employees and employers, so that governance can develop and evolve along with the wGT market, rather than behind it, and be based on data and evidence, rather than speculative hopes and fears.

## The appeal of genetic testing as a wellness benefit

In the US, employers often take keen interest in the health of their employees because most Americans receive their health insurance through their workplace^6^. Further, many employers are self-insured or self-funded, choosing to directly finance some or all of their employee health services and benefits instead of purchasing health insurance for employees, meaning that the employer is financially responsible for the healthcare claims of their employees. According to the 2020 Employer Health Benefits Survey published by the Kaiser Family Foundation, 67% of employees across all surveyed firms were in a self-funded plan. At firms with 5000 or more employees, 94% of employees were in a self-funded plan^7^. Furthermore, employer-sponsored health plans cover more than 157 million people in the US^8^.

Advances in genetic medicine have improved our ability to predict, prevent, and treat certain genetic conditions^9–11^. DTC testing, elective return of actionable “secondary” findings (e.g., hereditary cancers and certain cardiovascular conditions) following genomic testing according to American College of Medical Genetics and Genomics (ACMG) guidelines, and expanded efforts to engage family members after a patient’s genetic diagnosis (cascade screening) are increasing the numbers of individuals receiving genetic and genomic test results^12–14^. wGT could push these numbers higher, especially among generally younger and healthier populations who may not have cause or opportunity to pursue genetic testing through a clinical care infrastructure. Those who promote wGT suggest that such testing has the potential to lower employer healthcare costs as a result of: (1) employees becoming more informed about disease risks, making lifestyle changes, increasing preventive screenings or pursuing other interventions to reduce disease risks^15^; and (2) employers using aggregate employee test data to add other benefits that could potentially mitigate disease risks^16^.

A number of commercial vendors are marketing wGT services to employers^17^; however, it is currently unknown how many companies are actually offering wGT within their workplace wellness programs, what kind of testing is being offered, and whether the programs include services beyond mere testing, such as pre- or post-test genetic counseling. Further, little is known about employers’ motivations to provide wGT or how they access and utilize the resulting aggregate data. What we do know comes largely from press releases and media reports^18,19^. For example, executives at some US companies offering wGT have described this offering as “creating goodwill” with employees and being “a differentiator” that sets them apart from competing employers^20^. However, we have only limited data in the US or elsewhere on employee perspectives and concerns about wGT that would enable us to evaluate such claims^21^.

In a recent survey of employees at a biomedical research institution in the US, participants provided their views on wGT after being presented with three hypothetical scenarios for accessing genetic testing: (1) in the participant’s doctor’s office; (2) a workplace setting, and (3) via DTC testing. Of the 594 respondents, 70% of respondents indicated that they would prefer genetic testing in the workplace over a doctor’s office or DTC setting, though the survey did not solicit rationale. In addition, over 60% of respondents wanted to know about relevant laws and policies in place to protect their privacy and confidentiality^21^. It is important to note that the participants in this study were employed at a biomedical research institution that specifically focuses on genomics, and their views may not be representative of the broader population of employees’ views towards wGT. Further research is needed with employees at a range of diverse organizations. Previous research on employee perspectives regarding wGT found that employees have a desire to learn about their genetic information, and want a choice regarding who controls, stores, and has access to their genetic information^22^. For example, in one survey of university health center employees, respondents expressed strong interest in learning about their own genetic information while also rating privacy and confidentiality protections as very important^22^. The participants also indicated that including this information in their employee health records and insurance files was unacceptable, as was using their identifiable information in genetic research^22^.

Additionally, a 2017 Wamberg Genomic Consumer Survey of 536 US consumers with employer-sponsored health insurance reported that 65% of employees would be interested in genetic testing if their employers offered easy and affordable access with strict privacy and data ownership control, allowing test result access only to the employee and their doctor^23^. This latter stipulation underscores the privacy concerns employees have about wGT. Regardless of context, it remains the case that many people and policies, including the Genetic Information Nondiscrimination Act (GINA), regard genetic information as a unique category of health information, in that it is intrinsic, predictive, probabilistic, and has relevance for close genetic relatives^24^. In part due to these characteristics, policies providing consumer protection specific to genetic information enjoy broad support^25^.

## Historical perspectives on genetic testing in the workplace

While wGT is a relatively new phenomenon, employer use of genetic testing and screening (and associated ethical analysis of the practice) has been ongoing since at least the 1990s, with an initial focus on genetic monitoring and genetic vulnerability screening to mitigate employee risk to workplace hazards^6,26–28^. While the terms are sometimes used interchangeably, genetic monitoring includes frequent employee testing to determine the effects of toxic substances on employee health (e.g., DNA testing to measure carcinogen exposure to assess cancer risk)^29^, whereas genetic vulnerability screening includes testing an unselected group of employees once to identify workers with genetic susceptibilities that might make them more vulnerable to harm in the workplace (e.g., G6PD deficiency that increases sensitivity to oxidative stress, or sickle cell carriers who face increased risk in hypoxic environments)^26,27^. Since these very early experiences, concerns have been raised about the ELSI of such testing^30^, and these potential issues have increased with the expansion of genetic knowledge gained over the intervening decades.

A main ethical concern raised by historical workplace genetic vulnerability screening is that while employers argued that this screening could enable them to safeguard against workplace-related illness, it might also enable them to avoid legal liability for illnesses resulting from workplace hazards that could be attributed to an employee’s genetic predisposition^26^. Additionally, due to the newfound knowledge of genetic causes for some diseases, ethicists also raised the concern that this information could be used to discriminate against historically marginalized populations (e.g., by mandating sickle cell testing), and that a positive screening result could both facilitate discrimination and exacerbate stigma^28^. Historically, some organizations, including parts of the US military, tested individuals for sickle cell trait (SCT), for example, to restrict access to certain kinds of military service and benefits for individuals found to have the trait. However, due to conflicting evidence on the relationship between SCT and job-related risks, and concerns about genetic and racial discrimination, the testing as originally practiced was discontinued^31^. Furthermore, potential false positive and false negative screening results (the latter of which were more common in early genetic screening/testing) exacerbated these concerns^28^.

Early discussions of genetic vulnerability screening in the workplace raised the possibility that such screening could be completed as part of the pre-employment hiring process, similar to pre-employment use of other medical information and testing. The Americans with Disabilities Act (ADA), which was passed in 1990, regulates pre-employment medical testing in order to limit the potential for discrimination^32^. Several key legal cases have demonstrated the potential for discrimination and unethical use of screening for genetic conditions in the workplace^6^. In *Norman-Bloodsaw v. Lawrence Berkeley Labs* (1998), employees sued their employer for medical testing of employee blood and urine samples without their knowledge or consent. In addition to broad syphilis testing of employees, Black employees had been tested for sickle cell carrier status, and women had been tested for pregnancy. The 9th Circuit Court of Appeals reversed summary judgment for the defendants on the grounds of the 4th Amendment’s prohibition of unreasonable searches and seizures. Additionally, the court held that Title VII of the Civil Rights Act (which prohibits employment discrimination based on race, color, religion, sex, and national origin), allowed the plaintiff’s claims that they should not be forced to undergo genetic testing by an employer during the pre-screening process^33^. Perhaps the most prominent case is the Equal Employment Opportunity Commission’s (EEOC) 2001 suit against the Burlington Northern Railway Company alleging violations of the ADA^34–36^. Burlington Northern had been requiring blood samples from all employees who claimed they developed work-related carpal tunnel syndrome to determine whether they had a rare genetic predisposition to this condition, a finding that might help the employer avoid worker compensation claims costs. However, the company did not tell their employees that their samples would be used for genetic testing^6,27,29,37^. This case settled for $2.2 million^38^ and demonstrates the financial risk employers may face for inappropriate use of genetic testing. While this is critical background for a discussion of genetic and genomic testing in the workplace, it is important to note that how we use the language of wGT has changed dramatically since this early experience.

## Genetic testing within workplace wellness programs

In the 1970s, employers began offering workplace wellness programs promoting healthy behaviors among their employees^39^ to reduce health costs and to improve employee well-being and work productivity. The Health Insurance Portability and Accountability Act (HIPAA) in 1996 and Affordable Care Act (ACA) in 2010 set rules allowing for the use of workplace wellness programs. While both these laws prohibit health plans from adjusting premiums based on health status, they have an exception for wellness programs. The laws arguably encourage wellness programs by allowing employers to incentivize employee participation. After the implementation of the ACA, some wellness programs offer incentives or impose penalties that are equal to 30% of the cost of health insurance premiums under the employer’s plan, and as high as 50% for smokers. This can translate to the gain or loss of thousands of US dollars for individuals and families alike^16,40^, raising concerns that these programs can be coercive^41^.

wGT could fit well into workplace wellness programs since genetic and genomic testing may offer employees the opportunity to identify genetic risks and mitigate or prevent future disease (and potentially, associated healthcare costs). Indeed, a growing number of employers are offering, or considering offering, wGT as part of their wellness programs^17,20^. However, wGT brings with it both previously identified and new ELSI concerns. In response, some ELSI researchers focusing on genetic testing in the workplace have shifted their work from genetic monitoring and vulnerability screening to employee workplace wellness programs^42^. Given that the US differs with respect to the structure of health insurance and healthcare as compared to other countries, many issues in this space are of unique concern in the US. Differences such as the lack of a national health system, very high healthcare costs, the fragmentation of rules of healthcare regulation, and the distribution of insurers and employers across a large number of states, shape risks, benefits, and incentives for such programs.

Beyond the narrow category of workplace wellness programs that offer genetic testing, workplace wellness programs generally inspire critique. While many employers argue that workplace wellness programs can lead to healthier employees and lower costs for employers, this is not necessarily supported by currently-available data^41,43^. Studies and critiques of these programs have suggested that they do not address key employer concerns such as absenteeism^44^, healthcare coverage costs, or unhealthy employee behavior^38,45^, but rather serve to shift the cost of healthcare coverage from employers to employees^46^ by establishing rewards/penalties linked to health insurance premium levels. Additionally, there is worry that employees may forgo any concerns about the long-term risks associated with wellness programs (e.g., related to privacy and discrimination) in order to benefit from near-term financial incentives or to avoid penalties. Many of these overall concerns raised by workplace wellness programs could also apply to wellness programs that include wGT.

One issue unique to wGT relates to genetic privacy. The public are wary of potential misuse of their genetic information—public polling in 2018 revealed that 47% of respondents who had undergone DTC genetic testing (or whose family members had done so) expressed concerns about genetic privacy^47^. Genetic testing within an employment setting itself could potentially heighten these privacy concerns if there is not adequate protection for employees’ information and employment^26^.

## Legal landscape

In 2008, the US Congress passed GINA, a law prohibiting employers from using genetic information, broadly defined, for decisions regarding hiring, firing, promotion, or length of employment^48^. The law also prohibits covered health insurers from setting eligibility and premium or contribution amounts for health insurance based on genetic information. It does not, however, provide protections in the contexts of disability, long-term care, or life insurance^27^. Notably, GINA prohibits employers and health insurers from collecting genetic information; however, there are exceptions to this rule for certain cases, including monitoring one’s genetic information for changes due to exposure of toxic substances in the workplace and for voluntary workplace wellness programs^49^. Current GINA regulations generally state that a program is only voluntary if there is no incentive or penalty associated with providing genetic information to the employer^50^. Additionally, GINA only allows employers to access aggregate genetic information (rather than individual-level data) collected from employees in the wellness program. However, as is discussed further below, there have been recent efforts by regulators and Congress alike to alter this rule. While there are several other federal laws and policies that provide protections for employees in the context of voluntary workplace wellness programs, they are not specific to one’s private genetic information (see Table 1) (e.g., ADA, HIPAA, etc.)^33,51^.

**Table 1:** US laws regulating workplace wellness programs.

Law	Legal protections
Health Information Portability and Accountability Act (HIPAA) (1996)	● HIPAA’s Privacy Rule safeguards the privacy of protected health information, but only covers healthcare providers, health plans, health care clearinghouses and their business associates. If the employer’s health plan is administering the workplace wellness program, HIPAA’s Privacy Rule would apply ● Another part of HIPAA creates antidiscrimination laws related to setting premiums on the basis of health status (discussed further under the ACA)
Affordable Care Act (ACA) (2010)	● The ACA amended HIPAA to alter its antidiscrimination protections ● Together, the laws prohibit group and individual health plans from considering an individual’s health status when setting premiums; however, there is an exception for wellness programs ● The ACA set the allowable amount wellness programs can leverage as an incentive/penalty for participation at 30% of the cost of the health plan (50% for smoking cessation programs) ● The ACA and HIPAA set other rules for wellness programs, depending on the type of program, like who it must be offered to and how often a participant must be able to qualify for an incentive
Americans with Disabilities Act (ADA) (1990)	● The ADA restricts an employer from collecting medical information about an employee, with some exceptions ● One exception is for *voluntary* wellness programs ● *Voluntary* is not defined by law and there has been debate about what the rules regarding allowable incentives should be
Genetic Information Nondiscrimination Act (GINA) (2008)	● GINA prohibits employers from collecting genetic information, with some exceptions ● One exception is for *voluntary* wellness programs ● However, unlike the ADA, GINA defined *voluntary* to state that no incentives could be offered to provide genetic information ● GINA prohibits employers from accessing individual-level genetic data in wellness programs; they can only access aggregate data

Currently, it is unclear whether the complex web of federal and state laws that regulate medical information privacy, discrimination, and group health insurance coverage in employment—such as GINA, the ACA, the ADA, and HIPAA—actually ensure employee protections against inappropriate use or misuse of wGT by employers and insurance carriers^25,52,53^, such as accessing individual test results to inform employment decisions or increase life insurance premiums due to genetic predispositions.

Efforts have been made to put legal guardrails around the use of genetic information in employment and insurance markets to provide some protections for individuals^40^. However, many commentators feel these efforts have been inconsistent and inadequate^27^. The international landscape is unclear, as there is little relevant literature to date on wGT as described in this paper, and what literature exists is not recent^54^.

## Emerging policies

While experts recognize the potential benefits of wGT (e.g., testing for clinically-actionable mutations) for the employers’ overall preventive health strategy^55^, it is not yet clear if the benefits outweigh the risks for employees. In the absence of federal regulation providing guidance for employers or protections for employees there is some support for voluntary guidelines^6,42^ regarding implementation of wGT. For example, a 2010 statement by the American College of Occupational and Environmental Medicine (ACOEM) on Genetic Screening in the Workplace, argues that while genetic testing of the sort being offered through wGT has the potential to inform prevention and treatment approaches by way of pharmacogenomic, susceptibility, and diagnostic testing, it must be paired with guiding ethical and legal principles, scientific validity of wGT tests, and discussion of results with a trained health professional^56^.

Congress waded into the debate in 2017, with the introduction of HR 1313^57^. This bill would have allowed employers to incentivize or penalize employees for their decisions to disclose genetic information in a wellness program. The allowable incentive or penalty would have been up to 30% of health coverage costs, thus mirroring current incentives allowed by the ACA for general wellness programs. Additionally, it arguably would have allowed employers to collect individual-level data about employees’ private genetic or medical information within the context of a voluntary wellness program^58^. This would have thus significantly diminished employees’ genetic privacy rights. Professional societies such as the American Society of Human Genetics (ASHG) and the ACMG opposed this bill that sought to exempt workplace wellness programs from GINA and the ADA^59,60^.

Finally, the EEOC under the Trump Administration proposed rules that would have altered the protections of GINA in regard to workplace wellness programs^61^. For example, the proposed rules would have allowed rewards to incentivize individuals to share their genetic information, thus increasing concerns about coercion and the risks of breaches of privacy and the possibility of discrimination for employees. Of note, employers would not be required to share the type of employee health information they would be privy to or how they would use the information.

However, as of July 2021, any further action on these proposed rules has been paused by the Biden Administration^62^.

## The need for further ELSI research on wGT

While two existing commentaries discuss wGT^17,63^ and address many of the concerns highlighted above^55,64^, they focus primarily on the vendors offering the testing and the employers contracting with them. One of these, Stakeholders Assessing Genetics with Employers (SAGE), is a 2019 federally-funded project that provides some framework and context for conducting research on wGT^63^ and created a checklist for employers considering wGT. The four areas for consideration are: (1) Defining wellness program goals; (2) Specific types of genetic tests to be offered to employees; (3) Legal and policy considerations to mitigate liability risks and choosing a reputable vendor; and (4) Types of evidence employers should request to ensure that their wGT goals are achieved. Ultimately, there are many questions to be answered and little published research exploring the intersection of workplace wellness programs and genetic testing^17,41,65^.

Given the expectation that wGT will become more prevalent in the future, there is an urgent need to understand its benefits and risks. There also remains a need for empirical data to address the concerns raised by wGT and potential policy safeguards. It will also be important to understand concrete issues such as whether there are differences in implementing wGT in different settings, including the academic, non-profit, and corporate settings, and within small versus large companies (e.g., in whether or not genetic counseling is offered or how aggregate data are accessed and used). Our group has undertaken an initial step to study the ELSI issues surrounding wGT in real world settings and to elicit the perspectives of a broader repertoire of stakeholders—including employees, employers, and other relevant groups—whose perspectives are necessary for a fully informed view of wGT’s risks and benefits. Our current NHGRI-funded mixed-methods study aims to address the gaps in understanding of the wGT landscape and provide much needed empirical data to inform a normative assessment of wGT.

Recognizing that existing studies on wellness programs report minimal short-term benefits and do not account for wGT, our study will provide a comprehensive assessment of wGT from both the employee and employer perspectives—advancing an evidence-based approach to policy and decision making in this area of genetic testing. By undertaking a multifaceted impartial analysis, we hope to provide foundational information and insights to inform decision making for wGT, including (if appropriate) considerations for its implementation, so as to maximize its positive impact on individuals, employers, and society alike, while minimizing harm.

## Conclusion

Genetic testing offered outside the clinical setting is affordable and accessible to a large proportion of the population. US employers are beginning to offer genetic testing to their employees as part of their voluntary workplace wellness benefits, though it is unclear how widespread this practice is or will become. While this practice is in its infancy, it is important to consider the implications of wGT for clinical care, including outreach to genetic counselors, clinical geneticists, clinical assessment, follow-up, and management. Today’s wGT through commercial third-party vendors is substantially different from the historical model focused on genetic monitoring and vulnerability screening. Given this shift, there are new ELSI and policy concerns that have arisen; however, there is little to no research currently being conducted to assess these concerns. At this time, it is still unclear whether Pandora’s Box of wGT will produce benefits or risks and for whom. Our hope is that our study and the work of others will begin to address this question while there is still time to shape the outcome. If wGT becomes more widespread, as we predict, it is critical to have conceptual and empirical analyses of the risks and benefits to inform policy recommendations designed to maximize positive impact on individual health, employers, and society alike, while minimizing harm.

## References

[1] Regalado, A. More than 26 million people have taken an at-home ancestry test. MIT Technology Review. https://www.technologyreview.com/2019/02/11/103446/more-than-26-million-people-have-taken-an-at-home-ancestry-test/ (2019).

[2] Ugalmugle, S. & Swain, R. Direct-to-consumer genetic testing market size projections—2028. Global Market Insights, Inc. https://www.gminsights.com/industry-analysis/direct-to-consumer-dtc-genetic-testing-market (2020).

[3] Khan R, Mittelman D (2018). Consumer genomics will change your life, whether you get tested or not. Genome Biol..

[4] National Academies of Sciences, Engineering, and Medicine. *Exploring the Current Landscape of Consumer Genomics: Proceedings of a Workshop* (The National Academies Press, 2020). 10.17226/25713. 32721146

[5] Mujtaba, B. G. & Cavico, F. *Corporate Wellness Programs: Implementation Challenges in the Modern American Workplace*. https://papers.ssrn.com/abstract=2352353 (2013). 10.15171/ijhpm.2013.36 PMC3937880 24596864

[6] Brandt-Rauf PW, Brandt-Rauf SI (2004). Genetic testing in the workplace: ethical, legal, and social implications. Annu. Rev. Public Health.

[7] 2020 Employer Health Benefits Survey—Section 10: Plan Funding. *KFF* https://www.kff.org/report-section/ehbs-2020-section-10-plan-funding/ (2020).

[8] Health Insurance Coverage of the Total Population. *KFF* https://www.kff.org/other/state-indicator/total-population/ (2020).

[9] McGuire AL (2020). The road ahead in genetics and genomics. Nat. Rev. Genet..

[10] Evans JP, Berg JS, Olshan AF, Magnuson T, Rimer BK (2013). We screen newborns, don’t we?: realizing the promise of public health genomics. Genet. Med..

[11] Feero WG, Wicklund CA, Veenstra D (2018). Precision medicine, genome sequencing, and improved population health. JAMA.

[12] Miller DT (2021). Recommendations for reporting of secondary findings in clinical exome and genome sequencing, 2021 update: a policy statement of the American College of Medical Genetics and Genomics (ACMG). Genet. Med..

[13] Miller DT (2021). Correction to: ACMG SF v3.0 list for reporting of secondary findings in clinical exome and genome sequencing: a policy statement of the American College of Medical Genetics and Genomics (ACMG). Genet. Med..

[14] Miller DT (2021). ACMG SF v3.0 list for reporting of secondary findings in clinical exome and genome sequencing: a policy statement of the American College of Medical Genetics and Genomics (ACMG). Genet. Med..

[15] Nielsen DE, Carere DA, Wang C, Roberts JS, Green RC (2017). Diet and exercise changes following direct-to-consumer personal genomic testing. BMC Med. Genomics.

[16] Steck MB (2018). Workplace wellness programs: educating patients and families about discrimination via disclosure of genetic information. Clin. J. Oncol. Nurs..

[17] McDonald WS (2020). Genetic testing and employer-sponsored wellness programs: an overview of current vendors, products, and practices. Mol. Genet. Genom. Med..

[18] Mayer, K. Cisco adds emphasizing fertility, adoption benefits. Employee Benefit News. https://www.benefitnews.com/news/cisco-adds-emphasizing-fertility-adoption-benefits.

[19] Color Genomics Introduces Benefits Program. *Business Wire* https://www.businesswire.com/news/home/20150930006272/en/Color-Genomics-Introduces-Benefits-Program (2015).

[20] Singer, N. Employees JUMP AT GENETIC TESting. Is THAT A GOOD THIng? The New York Times. https://www.nytimes.com/2018/04/15/technology/genetic-testing-employee-benefit.html (2018).

[21] Sanghavi K (2021). Employees’ views and ethical, legal, and social implications assessment of voluntary workplace genomic testing. Front. Genet..

[22] Roberts L (2005). Perspectives on use and protection of genetic information in work settings: results of a preliminary study. Soc. Sci. Med.

[23] Wamberg Genomic Advisors. 65% of employees would pay for genetic testing from their employer sponsored health account, Wamberg Genomic Survey. https://www.prnewswire.com/news-releases/65-of-employees-would-pay-for-genetic-testing-from-their-employer-sponsored-health-account-wamberg-genomic-survey-300560945.html.

[24] U.S. Equal Employment Opportunity Commission. Fact Sheet: Genetic Information Nondiscrimination Act. U.S. Equal Employment Opportunity Commission. https://www.eeoc.gov/laws/guidance/fact-sheet-genetic-information-nondiscrimination-act. 10.1089/gtmb.2015.29002.bjk 26172622

[25] Murray T (2019). Is genetic exceptionalism past its sell-by data? On genomic diaries, context, and content. Am. J. Bioeth..

[26] Lemmens T (1997). ‘What about your genes?’ ethical, legal, and policy dimensions of genetics in the workplace. Politics Life Sci..

[27] Brandt-Rauf SI, Brandt-Rauf E, Gershon R, Brandt-Rauf PW (2011). The differing perspectives of workers and occupational medicine physicians on the ethical, legal and social issues of genetic testing in the workplace. N. Solut..

[28] MacDonald C, Williams-Jones B (2002). Ethics and genetics: susceptibility testing in the workplace. J. Bus. Ethics.

[29] Geppert CMA, Roberts LW (2005). Ethical issues in the use of genetic information in the workplace: a review of recent developments. Curr. Opin. Psychiatry.

[30] Dabney B (1981). The role of human genetic monitoring in the workplace. J. Occup. Med..

[31] De Castro M (2016). Genomic medicine in the military. npj Genom. Med..

[32] *Americans with Disabilities Act*. *42* (1990).

[33] Norman-Bloodsaw v. Lawrence Berkeley Lab, 135 F.3d 1260. 11648435

[34] Roberts LW, Barry LK, Warner TD (2011). Potential workplace discrimination based on genetic predisposition: views of workers. AJOB Prim. Res..

[35] Gottlieb S (2001). US employer agrees to stop genetic testing. BMJ.

[36] Roberts LW, Warner TD, Geppert CMA, Rogers M, Green Hammond KA (2005). Employees’ perspectives on ethically important aspects of genetic research participation: a pilot study. Compr. Psychiatry.

[37] Brandt-Rauf, S. I., Brandt-Rauf, E., Gershon, R., Li, Y. & Brandt-Rouf, P. W. Genes, jobs, and justice: occupational medicine physicians and the ethical, legal, and social issues of genetic testing in the workplace. *337699* https://repository.library.georgetown.edu/handle/10822/516139 (2011). 10.2190/NS.21.1.j 21411427

[38] EEOC AND BNSF settle genetic testing case under Americans with disabilities ACT. U.S. Equal Employment Opportunity Commission. https://www.eeoc.gov/newsroom/eeoc-and-bnsf-settle-genetic-testing-case-under-americans-disabilities-act-0.

[39] McIntyre A, Bagley N, Frakt A, Carroll A (2017). The dubious empirical and legal foundations of wellness programs. Health Matrix.: J. Law-Med..

[40] Oliphant EN, Terry SF (2016). GINA and ADA: new rule seriously dents previous protections. Genet. Testing Mol. Biomarkers.

[41] Hendricks-Sturrup RM, Cerminara KL, Lu CY (2020). A qualitative study to develop a privacy and nondiscrimination best practice framework for personalized wellness programs. J Pers. Med..

[42] Hui SA, Engelman K, Shireman T, Hunt S, Ellerbeck E (2012). Opportunities for cancer prevention using employee wellness programs. Am. J. Health Educ..

[43] Hull G, Pasquale F (2018). Toward a critical theory of corporate wellness. BioSocieties.

[44] Song Z, Baicker K (2019). Effect of a workplace wellness program on employee health and economic outcomes: a randomized clinical trial. JAMA.

[45] Lewis A (2017). The outcomes, economics, and ethics of the workplace wellness industry. Health Matrix.: J. Law-Med..

[46] Frakt, A. & Carrol, A. Do workplace wellness programs work? usually not—The New York Times. https://www.nytimes.com/2014/09/12/upshot/do-workplace-wellness-programs-work-usually-not.html?_r=0 (2014).

[47] POLL: Genealogical curiosity is a top reason for dna tests; privacy a concern. *NPR.org* https://www.npr.org/sections/health-shots/2018/06/01/616126056/poll-genealogical-curiosity-is-a-top-reason-for-dna-tests-privacy-a-concern.

[48] H.R. 493—110th Congress: Genetic Information Nondiscrimination Act of 2008.

[49] *42* U.S. Code *§ 2000ff–1(b)(2), (5)*.

[50] 29 C.F.R. § *1635.8(b)(2)(iii)*.

[51] Madison KM (2016). The risks of using workplace wellness programs to foster a culture of health. Health Aff..

[52] Drabiak K (2017). Caveat emptor: how the intersection of big data and consumer genomics exponentially increases information privacy risks. Health Matrix.: J. Law-Med..

[53] Jost, T. Workplace wellness programs: federal agencies weigh in (update on contraceptive coverage). Health Affairs Blog. 10.1377/hblog20150417.047125/full/.

[54] Soini S (2012). Genetic testing legislation in Western Europe—a fluctuating regulatory target. J. Community Genet..

[55] Murray MF (2018). A proposed approach for implementing genomics-based screening programs for healthy adults. NAM Perspect..

[56] Brandt-Rauf P, Borak J, Deubner DC, ACOEM Task Force on Genetic Screening. (2015). Genetic screening in the workplace. J. Occup. Environ. Med.

[57] H.R. 1313—115th Congress: Preserving Employee Wellness Programs Act of *2*017.

[58] Hudson KL, Pollitz K (2017). Undermining genetic privacy? employee wellness programs and the law. N. Engl. J. Med..

[59] ACMG signs letter expressing concerns about the use of genetic information included in the proposed preserving employee wellness programs act (H.R. 1313) (2017).

[60] ASHG Opposes H.R.1313, the Preserving Employee Wellness Programs Act. *ASHG* https://www.ashg.org/publications-news/press-releases/201703-hr1313/ (2017).

[61] EEOC Holds Remote Public Meeting on Wellness NPRM. U.S. Equal Employment Opportunity Commission. https://www.eeoc.gov/newsroom/eeoc-holds-remote-public-meeting-wellness-nprm.

[62] SHRM-SCP, L. N.-P., J. D. & SHRM-SCP, L. N.-P., J. D. EEOC Freezes Rules on Wellness Programs and Union ‘Official Time’. *SHRM* https://www.shrm.org/resourcesandtools/legal-and-compliance/employment-law/pages/eeoc-freezes-rules-on-wellness-programs-and-union-official-time.aspx (2021).

[63] Deverka, P. A. et al. StakeHolders Assessing Genetics with Employers (SAGE). (2020).

[64] National Academies of Sciences, E. *Implementing and Evaluating Genomic Screening Programs in Health Care Systems: Proceedings of a Workshop*. *Implementing and Evaluating Genomic Screening Programs in Health Care Systems: Proceedings of a Workshop* (National Academies Press (US), 2018). 29787046

[65] Lieberman, C. What wellness programs don’t do for workers. *Harvard Business Rev.* https://hbr.org/2019/08/what-wellness-programs-dont-do-for-workers (2019).

